# Plausible role for CHW peer support groups in increasing care-seeking in an integrated community case management project in Rwanda: a mixed methods evaluation

**DOI:** 10.9745/GHSP-D-14-00067

**Published:** 2014-08-31

**Authors:** Anne Langston, Jennifer Weiss, Justine Landegger, Thomas Pullum, Melanie Morrow, Melene Kabadege, Catherine Mugeni, Eric Sarriot

**Affiliations:** a International Rescue Committee, New York, NY., USA; b Concern Worldwide, New York, NY., USA; c ICF International, The Demographic and Health Surveys Program, Rockville, MD., USA; d World Relief, Baltimore, MD., USA; e Rwanda Ministry of Health, Kigali., Rwanda; f ICF International, Center for Design and Research on Sustainability, Calverton, MD., USA

## Abstract

**Background:**

The Kabeho Mwana project (2006–2011) supported the Rwanda Ministry of Health (MOH) in scaling up integrated community case management (iCCM) of childhood illness in 6 of Rwanda's 30 districts. The project trained and equipped community health workers (CHWs) according to national guidelines. In project districts, Kabeho Mwana staff also trained CHWs to conduct household-level health promotion and established supervision and reporting mechanisms through CHW peer support groups (PSGs) and quality improvement systems.

**Methods:**

The 2005 and 2010 Demographic and Health Surveys were re-analyzed to evaluate how project and non-project districts differed in terms of care-seeking for fever, diarrhea, and acute respiratory infection symptoms and related indicators. We developed a logit regression model, controlling for the timing of the first CHW training, with the district included as a fixed categorical effect. We also analyzed qualitative data from the final evaluation to examine factors that may have contributed to improved outcomes.

**Results:**

While there was notable improvement in care-seeking across all districts, care-seeking from any provider for each of the 3 conditions, and for all 3 combined, increased significantly more in the project districts. CHWs contributed a larger percentage of consultations in project districts (27%) than in non-project districts (12%). Qualitative data suggested that the PSG model was a valuable sub-level of CHW organization associated with improved CHW performance, supervision, and social capital.

**Conclusions:**

The iCCM model implemented by Kabeho Mwana resulted in greater improvements in care-seeking than those seen in the rest of the country. Intensive monitoring, collaborative supervision, community mobilization, and CHW PSGs contributed to this success. The PSGs were a unique contribution of the project, playing a critical role in improving care-seeking in project districts. Effective implementation of iCCM should therefore include CHW management and social support mechanisms. Finally, re-analysis of national survey data improved evaluation findings by providing impact estimates.

## Abstract (precis)

During national scale up of iCCM in Rwanda, greater improvements in care-seeking were found in the districts where Kabeho Mwana implemented its model than in the rest of the country. Success was attributed to an emphasis on routine data review, intensive monitoring, collaborative supervision, community mobilization, and, in particular, CHW peer support groups.

## INTRODUCTION

Integrated community case management (iCCM) is an equity-focused strategy designed to increase access to effective treatment for the leading causes of under-5 mortality by training and supporting front-line community health workers (CHWs) to identify and treat children for malaria, diarrhea, and pneumonia at the household level.^1^ This strategy has gained prominence on the global health agenda over the last decade. Over the past 2 decades, the Rwandan Ministry of Health (MOH) placed a strong emphasis on accessible care at the community level, holding the initial election of 12,000 volunteer CHWs in 1995. This was the start of a national community health strategy that led to the provision of iCCM to all communities. The Box highlights key benchmarks in the evolution of the Rwanda CHW and iCCM program.

**BOX. Milestones in iCCM Scale Up in Rwanda**

**1995:** National election of 12,000 volunteer community health workers (CHWs), with the initial task of community sensitization on preventive measures such as immunization, hygiene, and nutrition.

**2004:** Pilot of home-based management of malaria (HBM) in 3 districts.

**2005:** Second round of CHW elections, with 1 male and 1 female CHW (called *binômes*) elected from each village. HBM pilot expanded to 2 additional districts, and community case management of diarrhea with oral rehydration solution (ORS) and zinc piloted in 1 district.

**2006:** HBM scaled up to all 19 malaria endemic districts.

**2007:** MOH Community Health Desk established. Treatment of community case management for diarrhea with ORS and zinc approved.

**2008:** Treatment of acute respiratory infection (presumed pneumonia) with amoxicillin approved. National rollout of iCCM in initial 10 districts.

**2010:** iCCM scale up to all 30 districts.

Between 2005 and 2010 the Rwanda national iCCM program expanded and evolved into a robust community health network. CHWs received a 4-day training on iCCM, covering topics such as recognition and referral of danger signs, assessment and treatment, drug management, reporting, and community mobilization.^2^ CHWs were supervised by the health center-based community health in-charge as well as by cell coordinators, who were also CHWs and mainly responsible for supervising monthly reporting. CHWs were not paid for their services, but they did receive modest per diem for participation in official trainings. The main strategy for motivating the CHWs was a community performance-based financing (PBF) system, funded by government and donor resources, whereby CHWs were organized into cooperatives and received small payments into group accounts based on reporting and achievement of treatment and referral targets.^3^

## PROGRAM DESCRIPTION

In synergy with, and supporting, the evolution of MOH policies, 3 international nongovernmental organizations (NGOs) active in Rwanda—Concern Worldwide, the International Rescue Committee and World Relief—began implementing home-based management (HBM) in 2004. Building on the success of HBM, the 3 NGOs formed a consortium to implement the Kabeho Mwana (“Life for a Child”) Expanded Impact Child Survival Project from October 2006 to September 2011 with funding from the United States Agency for International Development Child Survival and Health Grants Program (CSHGP) and other donors. The project covered 6 districts in Rwanda—Gisagara, Kirehe, Ngoma, Nyamagabe, Nyamasheke, and Nyaruguru—representing one-fifth of all districts and about 1.9 million people, or 18% of the country's total population. These districts were predominantly rural (97% compared with the national average of 88%) with less access to health care: in 2010, 34.1% of women in the project districts reported serious problems accessing health services because of distance, compared with 24.2% in the non-project districts.^4^ (See Supplementary Tables 1 and 2). According to the statistics and the project budget of the Organization for Economic Cooperation and Development, Kabeho Mwana support represented 3.8% of the total health expenditure (government and partners) in Rwanda from 2006–2010.^5^^,^^6^

The purpose of the Kabeho Mwana project was to build the capacity of the MOH to roll out iCCM in line with national guidelines. To do so, Kabeho Mwana trained more than 6,100 CHWs in the national iCCM curriculum and provided tools required for the provision of iCCM, including a lockable box for storing drugs and supplies, respiratory timers, a spoon and cup for mixing oral rehydration solution (ORS), and treatment registers. The project supported the initial procurement of zinc, amoxicillin, ORS, and, later, rapid diagnostic tests for malaria and related supplies. These products then became part of the regular national supply chain, but the project continued to monitor stock levels and worked with district management to help prevent stock-outs.

Beyond training and equipping CHWs to implement iCCM consistent with the national guidelines, Kabeho Mwana established a strong presence with field offices in each district, allowing project staff to provide regular, intensive technical support to each of the MOH district offices and regular visits to each of the 88 health centers. Project interventions designed to strengthen the community health system included training key health center staff in iCCM supervision, with financial support in the first years of the project given directly to facilities to pay for supervision visits. Project staff also initiated quarterly data reviews with health center staff and the cell coordinators. All interventions and services were carried out through structures in the national health system.

Beyond training and equipping CHWs, the project provided intensive technical support to the MOH field offices.

The project prioritized community mobilization strategies designed to promote healthy behaviors and create demand for CHW services. Within the framework of the national health system, Kabeho Mwana introduced CHW peer support groups (PSGs), bringing together an average of 20 CHWs from 2 to 4 neighboring villages for monthly meetings. Peer support groups were designed primarily to support health promotion activities; however, they also served as fora for increased interaction between CHWs, encouraging problem-solving and mutual accountability. During group meetings, CHWs were trained in health promotion, guided through joint planning of home visits to deliver messages on healthy family practices and to monitor their adoption, and they worked together to compile monthly reports. CHWs received no material or financial incentives for attending PSG meetings and were also expected to perform their regular CHW functions.

**Figure:**
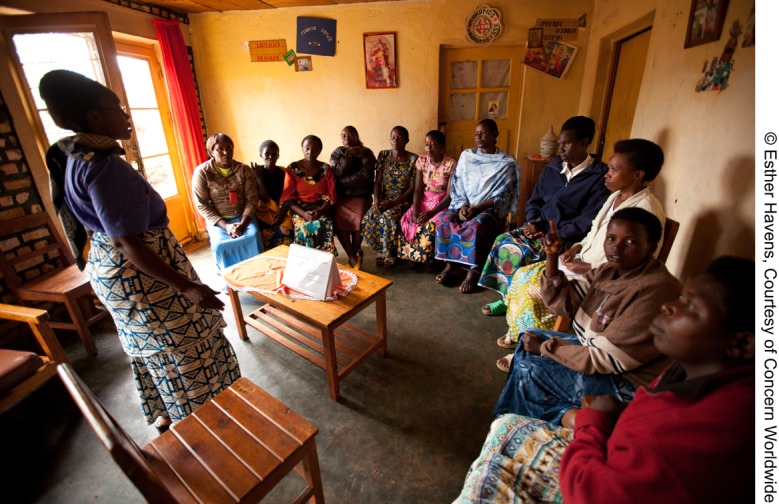
The Bahomwana CHW Peer Support Group meets in Gasambu village, Rwanda, to exchange ideas and challenges in order to accomplish and improve their work.

Three project promoters per district supported the formation and training of PSGs. Meeting facilitation was primarily the responsibility of the elected cell coordinators, who, under the supervision of the community health in-charge, were already tasked with collecting information from and supervising CHWs. The additional work of leading the PSGs was fairly limited; in fact, the meetings allowed cell coordinators time to collect, review, and discuss reports from all PSG members together at a single location, thus easing the work of reporting.

The Kabeho Mwana project final evaluation compared population health outcomes and community health service statistics in project districts with published national trends, which suggested a high level of achievement by the project.^6^ This sparked discussions between the MOH and partners and interest in the overall impact of the project approach, and whether achievements in the supported districts were attributable to the project or reflected improvements typical in all districts. The CHW PSG model implemented by Kabeho Mwana was identified as a unique element with the potential to provide critical motivation and quality control for CHWs. The nationwide scale of the Rwanda iCCM program created the opportunity for an innovative re-analysis of the DHS to examine the impact of the Kabeho Mwana project. If results were similar across the country then the Kabeho Mwana approach offered little benefit beyond the standard national iCCM package. However, if project districts had significantly outperformed non-project districts, then the MOH might choose to look more closely at the Kabeho Mwana approach and consider incorporating aspects of it, such as the PSG model, into its national program.

Peer support groups were identified as a way to provide critical quality control and motivation for CHWs.

## METHODS

This paper's primary method is re-analysis of the 2005 and 2010 DHS surveys to examine changes in care-seeking behavior for Kabeho Mwana project districts compared with districts without project support. This re-analysis is supplemented by the mixed-method external project final evaluation, conducted in August 2011, which we will turn to again to analyze factors that may have contributed to our findings.

### Analysis of How Project and Non-Project Districts Differed in DHS Estimates

We used data from 2 Demographic and Health Surveys (DHS) conducted in 2005^7^ and 2010,^4^ which approximately bracket the period of project implementation, to simulate a quasi-experimental design to evaluate the impact of the Kabeho Mwana project on care-seeking for diarrhea, symptoms of acute respiratory infection (ARI), and malaria. This analysis was possible because districts were the sampling strata used for the DHS. Both surveys used the standard DHS methodology for sampling^8^ and questionnaire^9^ construction, with some minor changes between the 2 surveys. In 2005, the sample size was 7,797 children 0–59 months, of whom 1,575 lived in project districts. In 2010, these figures were 8,605 and 1,780, respectively. Supplementary Table 3 presents the reported prevalence and the sample sizes by district for each year.

In both surveys, respondents were asked whether a child under 5 in the household had been ill in the past 14 days, the nature of the symptoms, and whether and what kind of health care provider was consulted. The definition for ARI symptoms changed between 2005 and 2010: In 2005, caregivers were asked about cough and rapid breathing, while in 2010 caregivers were asked about cough and rapid breathing that was chest-related and/or difficult breathing that was chest-related. From this information, we calculated rates of care-seeking for fever, diarrhea, and ARI symptoms, both from any provider (physician, nurse, or CHW) and specifically from a CHW. In addition, we included 3 non-project-specific maternal and child health indicators to determine whether there was a significant difference in the performance of project districts in health areas not related to iCCM (coverage of at least 3 antenatal visits, diphtheria, pertussis, and tetanus [DPT] immunization coverage, and vitamin A coverage). We also wanted to determine whether the focus of the project on iCCM might have inadvertently led to reduced performance in other health service areas.

We calculated the coverage rates for each of the indicators in 2005 and 2010 for all districts, for Kabeho Mwana project districts, and for non-project districts. The percentage increase in coverage in non-project districts was subtracted from the percentage increase in project districts to obtain the difference in the differences. We used logit regression to test whether the level of increase seen could be attributed to the project interventions or to other factors, controlling for the timing of the first CHW training in iCCM in each district and whether or not malaria was endemic in the district. (Supplementary Table 2 presents the basic characteristics of the districts included in the model.) The district was included as a fixed categorical effect to control for other unknown differences between the districts. The regression also included an interaction term coded “1” for the combination of the second survey and the project districts, and “0” otherwise. The coefficient of this interaction term will capture any additional use of services in the second survey and the project districts, beyond what would have been expected with an additive model.

Separately we calculated care-seeking rates by provider to compare the number of cases that consulted CHWs with the number of cases that consulted another trained provider at a government health facility, using a Pearson's chi-squared test to evaluate whether the difference between project and non-project districts was significant in each of the 2 years.

### Final Project Evaluation Methods

The final evaluation used a mixed-methods approach, including pre-post comparison of knowledge, practice, and coverage (KPC) surveys; process review data from project monitoring and the national health information system; qualitative methods (group and key informant interviews); and participatory engagement of the project team, including MOH staff. Indicators analyzed included the number of treatments given by CHWs per month, the number of home visits by CHWs per village per month, and the percentage of CHWs submitting complete reports to supervisors per month.

The project conducted 15 key informant interviews and 30 focus group discussions (FGDs) and analyzed observations from designers and implementers of the CHW PSG model. Sites for field visits, interviews, and FGDs were selected to represent all 6 districts equally and to purposively introduce some diversity and representativeness in the sample. Focus group discussions were held with mothers of children under 5 found at the health center, CHW members of the PSGs, cell coordinators, and members of CHW cooperatives. Interviews were conducted with community health in-charges, key project staff, the head of the National Community Health Desk, and hospital and district administrators. Interview and FGD transcripts were recorded, translated, and analyzed through thematic coding. Additional details on the evaluation methodology are included in the final evaluation report.^6^

## RESULTS

### Re-analysis of Rwanda Demographic and Health Surveys

Notable improvements in care-seeking for fever, diarrhea, and ARI symptoms occurred between 2005 and 2010 across all districts in Rwanda. However, the increases were significantly greater in the districts supported by Kabeho Mwana (Table 1). Care-seeking from any provider for all 3 conditions combined increased from 16% to 46% in the project districts, vs. 26% to 40% in non-project districts. The OR for care-seeking for each of the 3 conditions and for all 3 combined increased significantly more in project districts than non-project districts (adjusted OR for additional increase in use associated with project districts: fever OR = 2.54, *P*≤.001; diarrhea OR = 2.56, *P*≤.001; ARI OR = 2.35, *P*≤.01; combined OR = 2.24, *P*≤.001) (Table 2).

Care-seeking for fever, diarrhea, and acute respiratory infection improved across all districts, but especially in project districts.

**Table 1.** Care-Seeking for Fever, Diarrhea, and ARI Symptoms and Other MCH Interventions for Kabeho Mwana and Non-Kabeho Mwana Project Districts in 2005 and 2010

	**Non-KM Project Districts**			**KM Project Districts**			**Difference in the Differences**
	**2005**	**2010**	**Change**	**2005**	**2010**	**Change**	
	**N (%)**	**N (%)**	**%**	**N (%)**	**N (%)**	**%**	**%**
**Care-Seeking From Any Trained Provider**							
Diarrhea	803 (16.2)	808 (36.3)	+20.0	292 (8.4)	308 (40.0)	+31.6	+11.6***
Fever	1,485 (31.5)	1,003 (40.8)	+9.3	546 (20.3)	332 (48.7)	+28.4	+19.1***
ARI symptoms	964 (30.5)	233 (47.4)	+16.9	357 (20.9)	85 (57.9)	+37.0	+20.0**
All 3 conditions	2,551 (26.0)	1,570 (40.0)	+14.0	877 (16.3)	541 (46.0)	+29.7	+15.7***
**Non-Project Specific Indicators**							
At least 3 ANC visits in the most recent pregnancy	4,269 (13.7)	5,036 (36.2)	+22.5	1,115 (12.0)	1,274 (32.2)	+20.2	−2.3
Child 12–23 months received DPT3	1,271 (87.0)	1,262 (97.3)	+10.3	343 (88.5)	330 (95.7)	+7.2	−3.1
Child 6–59 months received vitamin A in past 6 months	5,461 (83.7)	6,168 (94.7)	+11.1	1,394 (85.8)	1,589 (85.6)	−0.2	−11.3

**Abbreviations:** ANC, antenatal care; ARI, acute respiratory infection; DPT3, diphteria, pertussis, and tetanus; KM, Kabeho Mwana; MCH, maternal and child health.

All of the tests are one-tailed. The tests in the last column of Table 1 correspond with the tests of the unadjusted odds ratios given in Table 2.

* *P*≤.05, ** *P*≤.01, *** *P*≤.001.

**Table 2.** Odds Ratios for Care-Seeking for Sick Children in Kabeho Mwana Project Districts vs. Non-Project Districts After Controlling for 2005–2010 Gains in Both Types of Districts

**Conditions**	**Unadjusted OR (95% CI)**	**Adjusted OR^a^ (95% CI)**
Diarrhea	2.47*** (1.47–4.15)	2.56*** (1.47–4.46)
Fever	2.49*** (1.66–3.73)	2.54*** (1.68–3.85)
ARI symptoms	2.53** (1.29–4.95)	2.35** (1.20–4.62)
All 3 conditions	2.31*** (1.66–3.21)	2.24*** (1.60–3.16)

**Abbreviations:** ARI, acute respiratory infection; CI, confidence interval; iCCM, integrated community case management; OR, odds ratio.

a Adjusted to control for the duration of time since implementation of iCCM, the district (as a fixed categorical effect), and whether malaria was endemic in the district. All of the tests are one-tailed.

* *P*≤.05, ** *P*≤.01, *** *P*≤.001.

We also looked specifically at care-seeking from CHWs. Care-seeking from CHWs for the 3 conditions combined was exceedingly low in both project and non-project districts in 2005 (1.2% and 0.6%, respectively). By 2010, it had risen to 12.4% in project districts and to 4.9% in non-project districts. As a result, the CHWs were contributing about one-quarter (27%) of the overall consultations in project districts compared with 12% in the non-project districts (Figure). Across all 3 iCCM conditions, CHWs in the districts supported by Kabeho Mwana were seeing a larger percentage of the cases than in other districts (Table 3).

**Figure.**
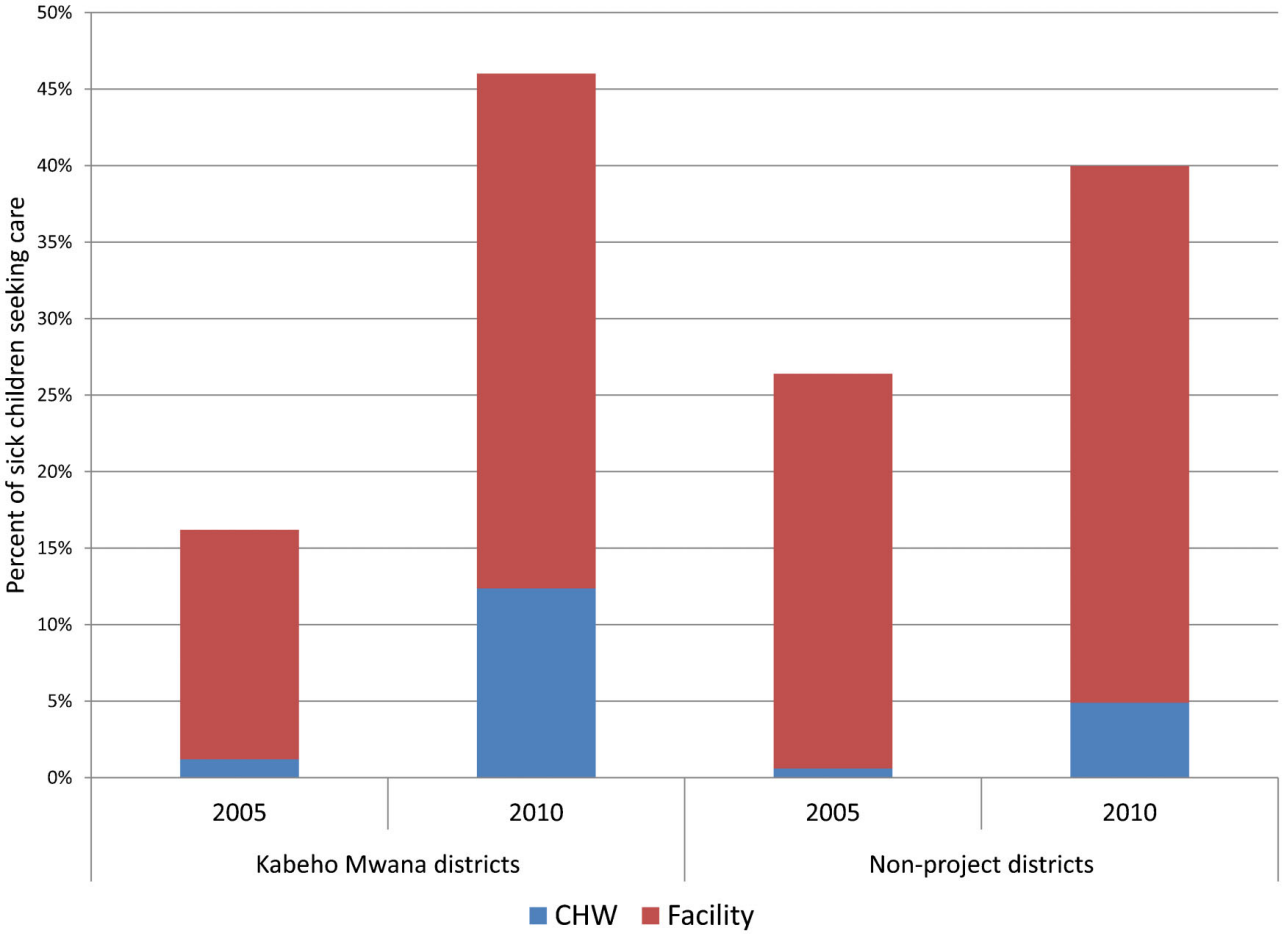
Care-Seeking From CHWs and Facilities for All iCCM Conditions in Children 0–59 Months, Kabeho Mwana Project vs. Non-Project Districts Abbreviations: CHWs, community health workers; iCCM, integrated community case management.

**Table 3.** CHW Contribution to Care-Seeking, Kabeho Mwana Project Districts vs. Non-Project Districts, 2005 and 2010

	**2005**		**2010**	
	**Non-KM Project Districts**	**KM Project Districts**	**Non-KM Project Districts**	**KM Project Districts**
**Diarrhea**	**n = 1,103**		**n = 1,132**	
Any provider	16.2 (13.3–19.1)	8.4*** (5.6–11.3)	36.2 (32.5–39.8)	40.0 (34.0–46.0)
CHW	0.2 (−0.2–0.60)	1.1 (0.1–2.1)	9.5 (7.3–11.7)	21.8*** (16.1–27.4)
Facility	16.2 (13.3–19.1)	8.4*** (5.6–11.3)	26.7 (23.0–30.3)	18.2* (13.6–22.9)
**Fever**	**n = 2,046**		**n = 1,355**	
Any provider	31.5 (28.7–34.3)	20.3*** (15.9–24.7)	40.8 (37.2–44.4)	48.7* (43.1–54.3)
CHW	0.4 (0.0–0.8)	1.2 (0.0–2.4)	12.1 (10.1–14.2)	26.1*** (21.0–31.3)
Facility	31.1 (28.3–33.9)	19.1*** (15.0–23.3)	28.6 (25.4–31.9)	22.5* (18.3–26.8)
**ARI Symptoms**	**n = 1,332**		**n = 322**	
Any provider	30.5 (27.2–33.8)	20.9** (16.3–25.5)	47.4 (39.2–55.7)	57.9 (45.9–69.9)
CHW	0.5 (0.0–0.9)	0.4 (−0.2–1.0)	7.7 (4.4–11.0)	27.5*** (16.6–38.3)
Facility	30.0 (26.8–33.2)	20.5** (15.8–25.1)	39.8 (31.8–47.7)	30.4 (20.8–40.1)
**All 3 Conditions**^a^	**n = 2,847**		**n = 2,142**	
Any provider	26.4 (24.1–27.6)	16.2*** (15.1–22.1)	40.0 (37.1–42.9)	46.0 (37.1–42.9)
CHW	0.6 (0.3–0.9)	1.2* (0.3–2.0)	4.9 (3.7–6.1)	12.4*** (8.9–15.9)
Facility	25.8 (23.5–28.1)	15.0*** (11.6–18.4)	35.1 (32.–38.1)	33.6 (29.3–38.0)

**Abbreviations:** ARI, acute respiratory infection; CHW, community health worker; KM, Kabeho Mwana.

a Includes children presenting with multiple conditions.

All data are shown as % (95% CI).

*P* values given are for the difference between rates of care-seeking in KM and non-KM districts in the same year.

* *P*≤.05, ** *P*≤.01, *** *P*≤.001.

Differences between the 2 surveys make it impossible to compare actual *treatment rates* between the 2 years for either fever or ARI symptoms. For diarrhea treatment with ORS or recommended home solution, the treatment levels increased nationally from 18.6% (95% confidence interval [CI] = 15.9–21.2) to 34.5% (CI = 31.4–37.6) with no significant difference between project and non-project districts. Current treatment guidelines include both ORS and zinc, but coverage for zinc, specifically, could not be measured.

The 3 health indicators not related to iCCM included in the analysis (coverage of at least 3 antenatal visits, DPT immunization coverage, and vitamin A coverage) all increased nationally with no significant difference between project and non-project districts.

### Results From the Final Project Evaluation

Findings, particularly from the FGDs and interviews, suggested that the PSG model served as a manageable and valuable sub-level of CHW organization that was associated with improved CHW performance, supervision, and increased social capital.

Findings suggest that the peer support group model helped improve CHW performance, supervision, and collaboration.

#### Performance and Coordination of CHWs

CHW productivity, reporting, motivation, and coordination were elements of CHW performance addressed through the PSG model according to those interviewed.

- **Productivity:** CHW productivity, as defined by home visits for health promotion activities and the number of treatments administered by CHWs, was greater in districts with PSGs than elsewhere. CHWs in Kabeho Mwana project districts averaged 44 visits per village per month, compared with 10–30 visits per village per month in a non-project district.^10^ On average, 356,387 home visits were conducted per quarter during the last 4 quarters of the project with beneficiary households receiving more than 2 visits per quarter. On the curative side, in the 12-month period prior to the project final evaluation, CHWs in project-supported districts provided one-third of all community treatments in Rwanda, while representing just 18% of the national target population for iCCM (personal communication, Cathy Mugeni and Erick Gajui; Rwanda MOH).
- **Reporting:** CHW reporting in project districts was high; 93% of CHWs submitted reports each month over the life of the project. While compensation from national PBF mechanisms likely played a role in the high levels of reporting, interviewees also suggested that regular interaction between CHWs and their supervisors during the PSG meetings eased the burden of work related to the compilation of reports, resolution of discrepancies, and timeliness. In contrast, the default structure of CHW cooperative meetings was not conducive to anything beyond data aggregation.
- **Motivation:** The energizing effect of joint planning among PSG members, according to FGD participants, kept CHWs engaged and committed to perform. Many CHWs reported being motivated to pool their resources to buy key household items, such as soap, in order to model target behaviors for their communities. The CHWs expressed their sense of being invested in the health of the households they covered. One PSG member explained, “It is a neighbor meeting a neighbor. They know each other. They know the local realities and education becomes easy.”
- **Collaboration:** The PSGs strengthened the role of the CHWs in linking the health facilities to the communities they served. A hospital director in Nyaruguru explained that CHWs are “our ambassadors in the community.” They “provide us with information which we can use to make decisions and transmit health messages to households; they serve as our representatives and guides.” A Maternal and Child Health Integrated Project (MCHIP) comparison assessment, which took place in the same period as the final evaluation and which evaluated iCCM activities in a non-project district, found that iCCM activities were better coordinated in project districts since there were mechanisms for peer support and the CHWs felt more accountable to the PSG.^10^

#### Supervision

In Rwanda, the community health in-charge was expected to visit each CHW to check drug stocks, ensure appropriate storage and drug management, and review the CHW register on a quarterly basis. In addition, the cell coordinator was expected to conduct peer supervision visits to all CHWs on a monthly basis. The position of cell coordinator was initially created by the project and ultimately scaled up nationally. Barriers to supervision by both the in-charges and cell coordinators were lack of sufficient time, transport, and human resources. Interviewees noted that PSGs lessened these barriers by enabling supervisor access to CHWs at an intermediate level between facility and community, during which they could address many supervision tasks within a small group setting. In addition, the PSGs provided an opportunity for informal peer supervision. Health facility personnel testified that this peer supervision helped to compensate for health facility staff limitations. The medical director of Nyamasheke District supported this assertion, “There is a difference since the establishment [of PSGs], and members train each other, self control, and do reports … you can follow up everyone regularly at lower cost.”

#### Social Capital

Participants in the FGDs explained that PSGs allowed for frequent interactions between members, experience sharing, and opportunities for cross-learning, ultimately resulting in a sense of camaraderie between members. The solidarity between CHWs also led to increased accountability, as no CHW wanted to under-perform. A cell coordinator in Ngoma District summarized the sense of collective and individual accountability in PSGs versus CHW cooperatives: “The more CHWs are present, the less they listen; but in a small group like the [PSG], their attention increases and they receive messages … things work well because there is a small group so results are more visible because everyone assesses their neighbor's performance.” Several cell coordinators cited examples where the PSGs voted to replace CHWs who were not committed and failed to model healthy behaviors.

In addition to accountability, the PSGs fostered a sense of trust, as demonstrated through the voluntary initiation and participation in rotating savings group activities. Group members contributed personal finances at each meeting, entrusting their investment to the group and to those approved to take out micro-loans. Kabeho Mwana did not provide any inputs for these activities; it was a group-initiated activity, thus underscoring its value to participants.

## DISCUSSION

Results of our analysis are twofold. First, care-seeking started from lower levels and increased significantly more in project districts than in non-project districts. Second, the contribution of CHWs to that increase was substantial. The contention that the approach used and the nature of the support provided under the Kabeho Mwana project was instrumental in attaining the greater improvements is supported by the lack of significant difference in the rates of increase of the 3 non-project indicators. It also suggests that the focus on iCCM under the Kabeho Mwana project did not have a negative impact on other aspects of health care.

Findings suggest that focusing on iCCM did not have a negative impact on other aspects of health care.

The Kabeho Mwana project was one of several actors supporting the MOH initiative to scale up iCCM from 2006 to 2011, and the core intervention of training and equipping CHWs to implement iCCM was not unique to the project. A variety of actors supported iCCM scale up in non-project districts, notably United Nations agencies, the National Malaria Control Program with support from the Global Fund, and other NGOs including World Vision, IntraHealth, and Management Sciences for Health. While specific data on the level of funding and nature of support for iCCM in the 24 non-project districts are unavailable, we assume that they varied between districts and consisted mainly of training and supplies to implement the standard national iCCM guidelines and structure. Kabeho Mwana achieved its mandate of providing technical support for iCCM scale up by going beyond simply training and equipping CHWs to implementing a series of interventions aimed at strengthening the overall health system, with a strong focus on CHW organization and supervision at the community level, all the while operating through the national health system without disproportionate financial expenditure.^5^^,^^6^

In addition to iCCM, all CHWs in Rwanda were trained in basic community mobilization and encouraged to do home visits, but the sole support structure developed by the national system was the supervision provided in principle by the health center community health in-charge (initially with financial support from the project). However, Kabeho Mwana also established PSGs to catalyze these health promotion activities, in addition to supporting the CHW curative role, and trained the cell coordinators to lead these groups. Integrating the curative function of the CHWs with frequent household visits promoted by the PSGs increased the visibility of CHW services, built the trust of the community, and may have led more mothers to recognize CHWs as an important source of care.

**Figure:**
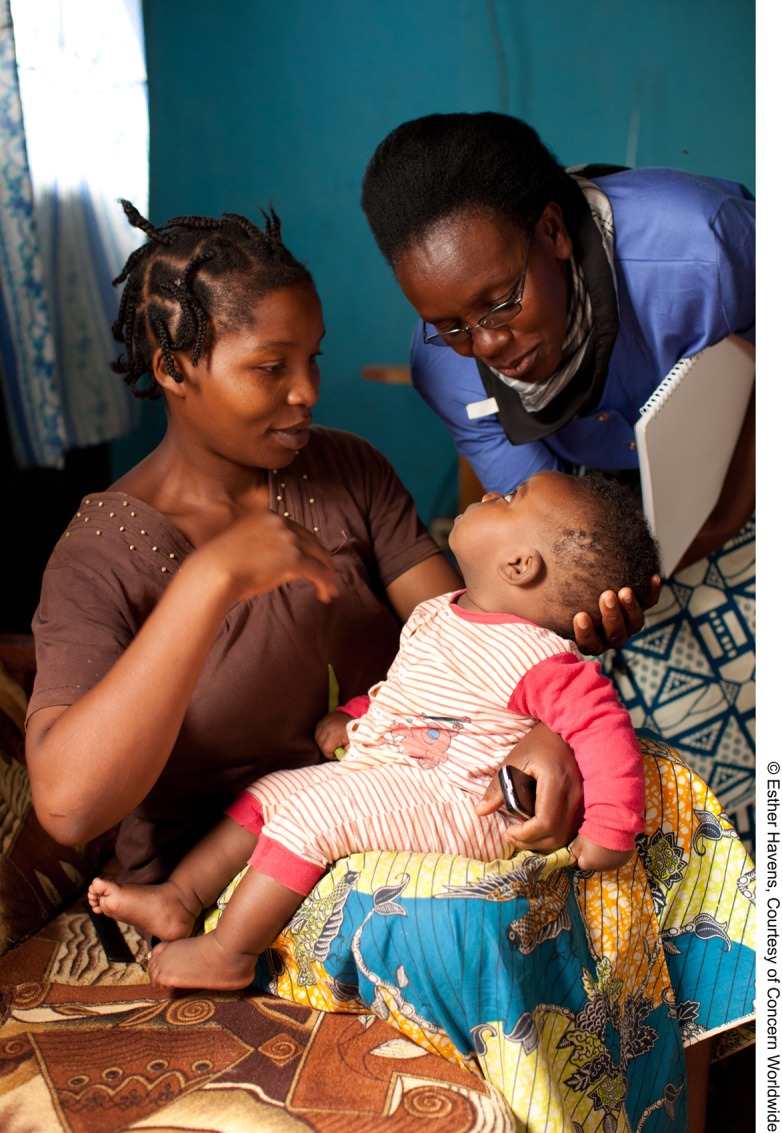
A community health worker and member of a Peer Support Group on a routine home visit.

The qualitative evidence demonstrates that, in addition to supporting community mobilization and prevention activities, the PSGs provided a critical mechanism to stimulate social capital and motivation among CHWs. The groups also facilitated CHW coordination, supervision, and reporting functions and increased interactions among and between CHWs and health center staff, leading to more effective community-facility linkages that may have contributed to the increase in care-seeking at the facility level.

Other aspects of the project may also have contributed to CHW performance. Quarterly data review meetings and increased supervision drew the attention of health facility staff and district management to the results reported by CHWs. This level of scrutiny may have motivated CHWs to increase their level of engagement with the community. We cannot differentiate the specific contributions of each element of support provided under the project; the PSGs should be considered as part of a comprehensive set of interventions to support community health and iCCM. However, our analysis suggests that PSGs—as the main intervention with CHWs who worked directly with the community—increased the visibility, accountability, and effective engagement of their members with the community, gaining the trust of care-givers of children under 5, and plausibly were a strong driver of increased care-seeking behaviors.

Peer groups may have driven increased care-seeking behavior by helping CHWs gain the trust of care-givers.

These findings support other evidence that has shown that peer support, specifically group meetings, may be an important contributing factor to CHW motivation.^11^ While the contribution of peer motivation, peer support, and peer accountability prompted by the PSGs cannot be quantified, they do appear to be fundamental motivators leading to a high level of CHW productivity. As a result, CHWs are highly engaged in their communities, beyond simply treating children. Home visits may contribute to increased demand for curative services at both the community and facility levels. A mixed-method impact evaluation from Uganda also showed that health promotion, including home visits by CHWs, improved care-seeking practices.^12^ The Kabeho Mwana model of the PSG was intended take advantage of these mechanisms to improve CHW performance.

Other studies have demonstrated the contribution of joint government–NGO partnerships implementing community-based strategies to higher coverage of key child health outcomes and reduced child mortality compared with concurrent sub-national secular trends as measured by the DHS.^13^ As documented by the *Lancet* series on child survival, projects such as Kabeho Mwana, that achieve high coverage of high-impact, evidence-based interventions related to sick-child care seeking and treatment also have a direct impact on under-5 mortality.^14^ Projects with similar implementation strategies and results in Mozambique and elsewhere have documented reductions in under-5 mortality of up to 62%.^15^

### Limitations

The DHS re-analysis provided an innovative complement to the pre-post mixed-method project evaluation. As an admittedly post-hoc exercise, however, it faced natural limitations.

Our assessment of integrated iCCM scale up does not capture all the variation in the implementation of iCCM across districts over time and with variable degrees of support, both internal and external. More data on CHW performance, supervision, and social capital in both project and non-project districts would have contributed significantly to this analysis, as would more information on the implementation of iCCM in other districts.

The analysis uses individuals as the unit of analysis but does not include individual-level covariates such as wealth or education. The main interest is in whether the change in care-seeking was different in the project districts than in the non-project areas—that is, in macro-level differences of differences—rather than in how the impact may have been different for different kinds of respondents.

Our analysis examines the change in care-seeking behavior. Actual treatment would have been a more proximal indicator for improvement in child health, but this was not possible because of differences between the 2 surveys. For ARI symptoms, treatment was not asked in 2005, and for fever, the addition of rapid diagnostic tests prior to data collection in 2010 makes it impossible to compare the results of the 2 surveys. The use of zinc for diarrhea was not asked in either survey. There was a change in the definition of ARI symptoms that results in a smaller number of more serious cases being included in the 2010 survey than was the case for 2005, which may account for some measure of the increase in care. The accuracy of care-giver reporting on ARI symptoms and its correlation to clinical pneumonia has been questioned by many,^16^ but in this case since we are comparing the difference in results for 2 groups within the surveys, we do not feel these concerns affect our conclusions.

The PSGs were unique to Kabeho Mwana districts and, as stated above, our analysis suggests that they may have contributed to improved CHW performance and ultimately to increased care-seeking. However, Kabeho Mwana implemented a comprehensive health systems strengthening approach to scale up iCCM, of which PSGs were just one element. All aspects of the project benefited from active engaged leadership, a flexible management style, skilled and dedicated Rwandan project staff, and enthusiastic collaboration from the MOH and local leaders. We cannot distinguish the specific effect of the PSG strategy on the overall performance of Kabeho Mwana districts. It is plausible that the main driver of improved outcomes in project districts was the difference in intensity of implementation support by Kabeho Mwana compared with other actors in non-project districts.

While a thorough treatment of the issue of sustainability is beyond the scope of this paper, the final evaluation and subsequent discussions with district and national leaders highlighted interest in scaling up the PSG model nationally. Enhancing CHWs' social capital, coordination, and peer support through a PSG approach could serve to sustainably strengthen the overall health system, and specifically community-level initiatives. However, this leads to some key questions about the cost of integrating PSGs into the national model. The cost to implement PSGs was not the subject of either the final evaluation or this analysis, and the cost if implemented by the MOH would likely differ from the cost as implemented by an externally funded NGO. Because the PSG model augments the existing strategy by adding a sub-level of organization, its costs should be manageable, but further examination to quantify those costs are needed to better inform policy decisions.

## CONCLUSION

Rwanda has achieved remarkable results in reducing child mortality. From a health systems strengthening perspective, while iCCM was being scaled up nationally in Rwanda, the Kabeho Mwana project placed additional emphasis on building community health systems and establishing and strengthening CHW supervision and peer support systems. These elements contributed to significantly greater improvements in care-seeking for fever, diarrhea, and ARI in districts supported by Kabeho Mwana as compared with other districts. While it is not surprising that an externally supported project resulted in improved care-seeking as compared with the existing national CHW strategy, these differences are now quantified and suggest that the approach used under Kabeho Mwana can be useful in improving CHW performance, and possibly improve the cost-effectiveness of performance-based financing.

Effective implementation of iCCM must go beyond training and equipping CHWs and include both overall health systems strengthening as well as CHW support mechanisms at the community level.^17^ The CHW PSG appears to have been an effective model for CHW peer support and motivation that enabled intensive population coverage of all households in the 6 districts while also providing a mechanism to integrate CHWs' preventive and curative functions and increased opportunities for supervision and reporting. Further testing of the PSG strategy for national scale up would require a quasi-experimental design, with attention to care-seeking and appropriate treatment. It should also focus on additional health promotion benefits and intermediary results in CHW supervision, motivation, and performance.

This article reports an innovative approach to estimating the effect of an iCCM intervention led jointly by 6 Rwandan health districts and a consortium of NGOs. From an evaluation perspective, DHS data proved useful in supplementing evaluation findings and elucidating the differential impact of iCCM under 2 implementation modalities. Countries and partners may seek to identify appropriate evaluation designs when sufficient clusters and enumeration areas can be included in the “intervention zone,” and timelines allow. This could enrich country evaluation models for large-scale innovations in service delivery.

## Notes

**Peer Reviewed**
